# Development of a Blocking ELISA Using a Monoclonal Antibody to a Dominant Epitope in Non-Structural Protein 3A of Foot-and-Mouth Disease Virus, as a Matching Test for a Negative-Marker Vaccine

**DOI:** 10.1371/journal.pone.0170560

**Published:** 2017-01-20

**Authors:** Yuanfang Fu, Pinghua Li, Yimei Cao, Na Wang, Pu Sun, Qian Shi, Xincheng Ji, Huifang Bao, Dong Li, Yingli Chen, Xingwen Bai, Xueqing Ma, Jing Zhang, Zengjun Lu, Zaixin Liu

**Affiliations:** 1 Lanzhou Veterinary Research Institute of Chinese Academy of Agriculture Science, State Key Laboratory of Veterinary Etiological Biology, National Foot-and-Mouth Disease Reference Laboratory, Key Laboratory of Animal Virology of Ministry of Agriculture, Xujiaping, Yanchangpu, Lanzhou, Gansu, China; 2 Xinjiang Entry-Exit Inspection and Quarantine Bureau, Urumqi, China; University of Melbourne, AUSTRALIA

## Abstract

Foot-and-mouth disease (FMD) is a devastating animal disease. Strategies for differentiation of infected from vaccinated animals (DIVA) remain very important for controlling disease. Development of an epitope-deleted marker vaccine and accompanying diagnostic method will improve the efficiency of DIVA. Here, a monoclonal antibody (Mab) was found to recognize a conserved “AEKNPLE” epitope spanning amino acids 109–115 of non-structural protein (NSP) 3A of foot-and-mouth disease virus (FMDV; O/Tibet/CHA/99 strain), which could be deleted by a reverse-genetic procedure. In addition, a blocking ELISA was developed based on this Mab against NSP 3A, which could serve as a matching test for a negative-marker vaccine. The criterion of this blocking ELISA was determined by detecting panels of sera from different origins. The serum samples with a percentage inhibition (PI) equal or greater than 50% were considered to be from infected animals, and those with <50% PI were considered to be from non-infected animals. This test showed similar performance when compared with other 2 blocking ELISAs based on an anti-NSP 3B Mab. This is the first report of the DIVA test for an NSP antibody based on an Mab against the conserved and predominant “AEKNPLE” epitope in NSP 3A of FMDV.

## Introduction

Foot-and-mouth disease (FMD) is a highly contagious viral disease that affects many cloven-hoofed animals. The causative agent is the FMD virus (FMDV), which is a single-stranded, positive-sense RNA virus belonging to the *Aphthovirus* genus in the *Picornaviridae* family. The FMDV genome contains a single open reading frame (ORF) that encodes structural proteins (SPs; VP1, VP2, VP3, and VP4) and non-structural proteins (NSPs; L, 2A, 2B, 2C, 3A, 3B, 3C, and 3D). The NSPs are relatively conserved among 7 distinct FMDV serotypes [1, 2].

FMD control mainly relies upon vaccination with whole–virus inactivated vaccines and restricting the movement of infected animals in epidemics regions. Generally, the production of FMDV inactivated vaccine is required to remove NSPs before emulsification. Otherwise, the vaccine will interfere with DIVA test based on detecting NSP antibody, especially when animals have been repeatedly vaccinated with inactivated vaccines produced by poor purification procedures. Such an influence is obvious in milk-producing animals and breeding stocks. Detailed instructions on how to confirm the purity of vaccine antigen are published in the Terrestrial Manual for FMD (2012) from the World Organisation for Animal Health [3]. However, vaccine antigens purification will greatly increase production costs. Therefore, the development of epitope-deleted marker vaccine and a matching test will provide an alternative way to decrease vaccine costs and improve the efficacy of FMD control.

Identification of dominant epitopes that are conserved and deletable is the basis for developing a negative-marker virus and vaccine. Such epitopes can be found in FMDV NSP 3A, 3B, and 3D [4–6]. With modification of these epitopes, a negative-marker virus can be produced by a reverse-genetic operation [7–10]. The FMDV 3A protein is a partially conserved protein with 153 amino acids (aa), or 143 aa in the case of the Cathay topotype of type O FMDV. The C-terminal half of the NSP 3A protein could be partially deleted, and the recombinant viruses were attenuated to some extent [9–11]. A Cathay topotype of the type-O marker FMDV (r-HN/3A93–143) was recently generated in our lab by deleting residues 93 to 143 (a region harboring immunodominant B cell epitopes) in the NSP 3A protein. The marker virus can adapt to replicate in suspension culture in BHK21 cells, and the marker vaccine is effective in protecting pigs from challenge with the wild type (WT) virus [9].

In this study, a monoclonal antibody (Mab) against NSP 3A (designated 3A24) was produced by a traditional method. This Mab recognize a conserved and immunodominant epitope (^109^AEKNPLE^115^) in NSP 3A of O/Tibet/CHA/99. A blocking ELISA (3A-Mab-bELISA) was developed using Mab 3A24 as a detection antibody for DIVA diagnosis. This method can be used as a matching test for the marker vaccine made from the FMDV with deletion of residues 109 to 115 in the NSP 3A. A good overall concordance was observed between the 3A-Mab-bELISA and previously developed 3B-Mab-bELISA [12] and commercially available PrioCHECK^®^ FMDV NS ELISA. Therefore, the development of a blocking ELISA targeted to the conserved “AEKNPLE” epitope of NSP 3A will provides a suitable companion test for the marker vaccine to differentiate infected from vaccinated animals.

## Materials and Methods

### Recombinant proteins and polyclonal and monoclonal antibodies

The soluble recombinant proteins NSP 3A, 3B, 3D, and the NSP 2C epitope region protein in inclusion body form were expressed in BL21 (DE3) pLysS or Rosetta (DE3) pLacI strains of *Escherichia coli (E*. *coli)* cell, and purified with Ni-NTA His binding resin (Invitrogen, California, USA) according to the manufacturer’s instructions as described previously [13]. The fusion protein 2C3AB, which contains B-cell epitope regions of 2C (nucleotides 1–174 and 682–954 bp of the 2C coding region) and the whole NSP 3A and 3B proteins of FMDV was expressed in form of inclusion body in BL21 (DE3) pLysS *E*. *coli* cells, and purified using a HisTrap HP affinity chromatography column as described previously [14].

A polyclonal antibody against FMDV the NSP 2C epitope regions was produced by immunization of two rabbits with the purified prokaryotically expressed NSP 2C epitope region, as described previously [12]. A Mab against NSP 3A was produced by traditional methods [15], briefly, the prokaryotic-expressed NSP 3A used as an immunogen to immunize female Balb/c mice, the spleen cells of the mouse with the highest antibody were used to fuse with SP2/0 cells to preparation of hybridoma cell. The supernatants from the hybridomas were screened using a NSP 3A indirect ELISA. The positive hybridomas cells were inoculated into abdomen of mouse to produce ascitic fluid. The Mabs were purified from ascitic fluid by the Affi-Gel protein A (Amresco, OH, USA) and conjugated with peroxidase according to the instruction manual of EZ-Link Plus Activated Peroxidase kit (thermo, USA). The Mabs were isotyped using Mouse Monoclonal Antibody Isotyping Kit (Sigma, USA). The reactivity of Mabs with different recombinant NSPs (3A, 3B, 3ABC, 2C3AB, and 3D) was determined by an indirect ELISA [12]. The binding sites of Mabs were analyzed using 13 synthetic peptides corresponding to the aa sequence of FMDV-3A by a peptide ELISA [12].

### Peptides

All peptides were synthesized by Genscript, Inc. (Nanjing, China; Table 1). The synthetic peptides included 10 overlapping peptides corresponding to the aa sequence of FMDV-3A, which cover aa 71–143 of O/HN/CHA/93 (with high homology to O/GD/CHA/86; GenBank AJ131468). In addition, 3 peptides (Q1, Q3, and Q7) were synthesized based on matching aa sequences in FMDV O/Tibet/CHA/99 3A, which are absent or different from that in the Cathay topotype of type-O FMDV [4]. The purity of these peptides were ≥90%, as determined by high-performance liquid chromatography. All peptides were stored at -70°C in dimethyl sulfoxide as stock solutions at 5 mg/ml.

**Table 1:** Synthetic peptides used to identify specific B cell epitopes against FMDV 3A Mab.

Peptide	Amino acid sequence	Location	Reference strain
A1	NIVIMLREARKRRQS	71–85	O/HN/CHA/93
A2	LREARKRRQSVDDSL	76–90	O/HN/CHA/93
A3	DDDAALDDAEKNPLE	92–105	O/HN/CHA/93
A4	AALDDAEKNPLEASG	94–108	O/HN/CHA/93
A5	LEASGASAVGFRER	104–117	O/HN/CHA/93
A6	ASAVGFRERSPTEQK	109–123	O/HN/CHA/93
A7	ERSPTEQKTCDDVNT	116–130	O/HN/CHA/93
A8	EQKTCDDVNTEPVVP	121–135	O/HN/CHA/93
A9	CDDVNTEPVVPGREQ	125–139	O/HN/CHA/93
A10	EPVVPGREQPRAE	131–143	O/HN/CHA/93
Q1	NEYIEKANITTDDK	91–104	O/Tibet/CHA/99
Q3	TDDKTLDEAEKNPL	101–114	O/Tibet/CHA/99
Q7	ERTLPGHKTSDDVN	126–139	O/Tibet/CHA/99

### Identification of a Mab against a native epitope of NSP 3A

The binding ability of an anti-NSP 3A Mab to recombinant FMDV NSP 2C3AB was determined by performing a solid-phase blocking ELISA using sera from FMDV-infected animals as a blocking agent. The procedure used for the blocking ELISA had been described previously [12]. Positive serum samples derived from cattle, sheep, and swine infected with the O/CHA/99 strain of FMDV at 30 days post-infection (DPI), and negative serum samples derived from clinically healthy, unvaccinated cattle, sheep, and swine were used in the test. Results were expressed as percent inhibition (PI) using the following formula: PI = (1–[OD sample/OD negative control]) × 100%.

### Conservative analysis of B cell epitopes in different serotypes with various FMDV 3A aa sequences

The aa sequences of 12 FMDV reference strains were aligned to analyze the conservation of the NSP 3A protein. Their GenBank accession numbers are as follows: O/Tibet/CHA/99 (CAD62370.1), O/Akesu/58 (AAM44304.1), O/TAW/99 (CAD62208.1), O/TAW/97 (AAT01778.1), O/GD/ CHA/86 (AJ131468), O/GD/2010/s (O/Mya/98, pig origin, unpublished data), Asia 1/JS/CHA/2005 (ABM66095.1), Asia1/YS/CHA/2005 (ADU56664.1), Asia1/VN/LC04/2005 (ADC92544.1), A/IRQ/09 (AER28328.1), A/VIT/2004 (AEO16200.1), and A22/IRQ/64 (AAT01706.1). The 3A aa sequences were aligned using DNASTAR Lasergene 7.1 software (DNASTAR, Inc., Madison, WI, USA).

### Sera origins

A total of 787 cattle sera, 637 sheep sera, and 565 pig sera were used in this study. The sera groups were described as follow: (Ⅰ) sera from unvaccinated healthy animals, including 84 from cattle, 88 from sheep, and 88 from swine, which were obtained from an FMD-free region; (Ⅱ) sera from healthy vaccinated animals, including 99 from cattle and 176 from swine that had been inoculated twice with an inactivated vaccine against type O FMD; (Ⅲ) sera from infected animals, including 100 samples from cattle infected with Asia 1/JS/CHA/05 or O/Tibet/CHA/99 FMDV collected between 10–28 DPI; 88 serum samples from sheep infected with Asia 1/JS/CHA/05 collected between 11–365 DPI; 88 sera collected from pigs infected with O/Tibet/CHA/99 collected between 3–11 DPI; (Ⅳ) sera from experimentally infected animals, including 40 sera collected from 4 cattle infected with A/WH/CHA/2009 FMDV at 0–229 DPI; 48 sera collected from 2 sheep infected with O/Tibet/CHA/99 FMDV at 0–417 DPI; 16 sera collected from one pig infected with O/ Tibet/CHA/99 FMDV at 0–194 DPI. (Ⅴ)1074 field sera collected from animal herds suspected of FMDV infection in China during 2009–2014, including 464, 413, and 197 sera collected from vaccinated cattle, sheep, and pigs, with all serologically positive animals being slaughtered after surveillance; (Ⅵ) A total of 12 serum samples were obtained from 3 cattle and 3 pigs before and after inoculation with the wild type O/HN/CHA/93 (Cathay topotype) at 28 DPI; 16 serum samples were obtained from 3 cattle and 5 pigs before and after inoculation with the 3A epitope-deleted marker virus (r-HN/3A_93-143_) at 28 DPI [9]. All wild type virus-inoculated pigs show typical FMD signs, while all marker virus-infected pigs only showed low-level clinical signs. No cattle showed clear clinical signs after infection with the wild type virus (O/HN/CHA/93) or marker virus. and (Ⅶ) positive and weak positive controls that were derived from animals experimentally infected with type O or Asia 1 FMDV at 30 DPI, which showed different levels of antibody titers against both SPs and NSP 3ABC by a liquid phase blocking ELISA (LPB-ELISA) and a 3ABC indirect ELISA (3ABC-I-ELISA), which developed in our laboratory as described by Hamblin et al [16,17] and Lu et al [18]; Negative control sera used in test was derived from clinically healthy unvaccinated animals with antibodies against SPs of FMDV O, A and Asia 1 lower than 4 by LPB-ELISA and the antibody value against NSP 3ABC lower than 0.2 by 3ABC-I-ELISA.

### Development of the 3A-Mab-bELISA

The 3A-Mab-bELISA was performed as described previously, with some modifications [12]. Briefly, 96-well ELISA plates were coated with 4.0 μg/ml purified polyclonal antibody of 2C epitope protein diluted in carbonate buffer (pH 9.6) and incubated overnight at 4°C. After 3 washes with PBST (0.01 M PBS, 0.05% Tween20), purified 2C3AB fusion protein was diluted to an optimal concentration (1.0 μg/ml) in PBST, 100 μl/well was added, and the plate was incubated for 60 min at 37°C. After 3 washes, the serum samples were diluted 1:5 in dilution buffer (PBS containing 5% normal rabbit serum, 1% BSA, and 0.05% Tween-20) and incubated on a plate rocker overnight at room temperature (20–25°C). After 5 washes, the horseradish peroxidase (HRP)-conjugated 3A Mab (3A24) was added to the wells in the same dilution buffer at an appropriate working concentration (0.8 μg/ml) and incubated for 1 h at 37°C. After 5 washes, TMB substrate was added. After 10–15 min, 0.6 N H_2_SO_4_ was added to each well to stop the reaction. The OD value was measured at 450 nm. The PIs of the test samples (positive control, weak positive control, and samples) were calculated using the following formula: PI = (1–[test sample OD/negative control OD]) ×100%.

### Determination the cutoff criterion for the 3A-Mab-bELISA test

The cutoff value of the 3A-Mab-bELISA was determined by testing 811 sera from different origins. Serum samples from 535 uninfected animals (sera group Ⅰand Ⅱ) were used to estimate the diagnostic specificity, and 276 infected sera (sera group Ⅲ) were used to estimate the diagnostic sensitivity. The PI value for each sample was calculated according to the formula described above. The optimal cutoff value of the 3A-Mab-bELISA test was selected based on the distribution of PIs that yielded a relatively high sensitivity and specificity.

### Evaluating the performance of the 3A-Mab-bELISA test

After determining the cutoff criterion, panels of sera of different origins (sera group Ⅰ~Ⅳ) was used to evaluate the performance of 3A-Mab-bELISA test by comparison with the 3B-Mab-bELISA and PrioCHECK NS ELISA tests (Prionics AG, Schlieren-Zurich, Switzerland). The results of 3B-Mab-bELISA and PrioCHECK NS ELISA tests for sera group Ⅳ referred to previous publication [12]. In addition, 1074 field sera (sera group Ⅴ) were tested by above three tests to study their concordance by receiver operating characteristic (ROC) curve analysis.

### ROC analysis

The ROC analysis was performed by the Sigmaplot software using the method described by DeLong et al [19]. This method provides a statistical comparison of the areas under the curves for the various ELISAs. ROC curve area differences were analyzed by the McNemar’s Chi Square test (S-Plus) to show the differences of three ELISAs. For the set the status of field samples, the sample was considered to be from an FMDV infected animal when any two or all three tests gave positive results, otherwise the sample was considered to be from an uninfected animal.

The 3B-Mab-bELISA test was developed previously in our laboratory [12]. This test is based on a Mab against a conserved and immunodominant epitope (^24^ GPYAGPMER ^32^) in NSP 3B of FMDV. The PrioCHECK FMDV NS ELISA Kit was obtained commercially (Prionics, Schlieren-Zurich, Switzerland). Both tests employed a blocking-ELISA format and the results were expressed as the PI rate compared with the negative control. Serum samples with a PI ≥50% were considered to be from infected animals, and serum samples with a PI rate <50% were considered to be from non-infected animals. The manufacturers’ instructions were strictly followed for each test.

### Detection the serum samples from animals infected with 3A epitope-deleted marker virus by the 3A-Mab-bELISA test

The 3A-Mab-bELISA, 3B-Mab-bELISA, and PrioCHECK NS ELISA tests were used simultaneously to detect serum samples from animals infected with the NSP 3A epitope-deleted marker virus and the wild type virus(sera group Ⅵ), in order to evaluate the potential of 3A-Mab-bELISA as a companion test for the negative-marker vaccine of the 3A epitope-deleted FMDV.

### Compliance with ethical standards

All animal experiments related to infection of FMDV were performed in Biosafety Level 3 facilities in Lanzhou Veterinary Research Institute (LVRI). The rules described by the Animal Ethics Procedures and Guidelines of the People’s Republic of China were strictly complied with during animal infection experiments and rabbit or mouse antibody preparation. All these experiments were also approved by the Animal Ethics Committee of LVRI, Chinese Academy of Agricultural Sciences (Permission number: SYXK-GAN-2010-0001). All animals used in the present study were acclimatized for one week before the beginning of the experiment, and the animals were euthanized by exsanguination under deep anesthesia (intramuscular injection of chlorpromazine at 2–6 mg/kg) at the end of the experiment.

## Results

### Preparation and identification of Mabs

**Fig 1:**
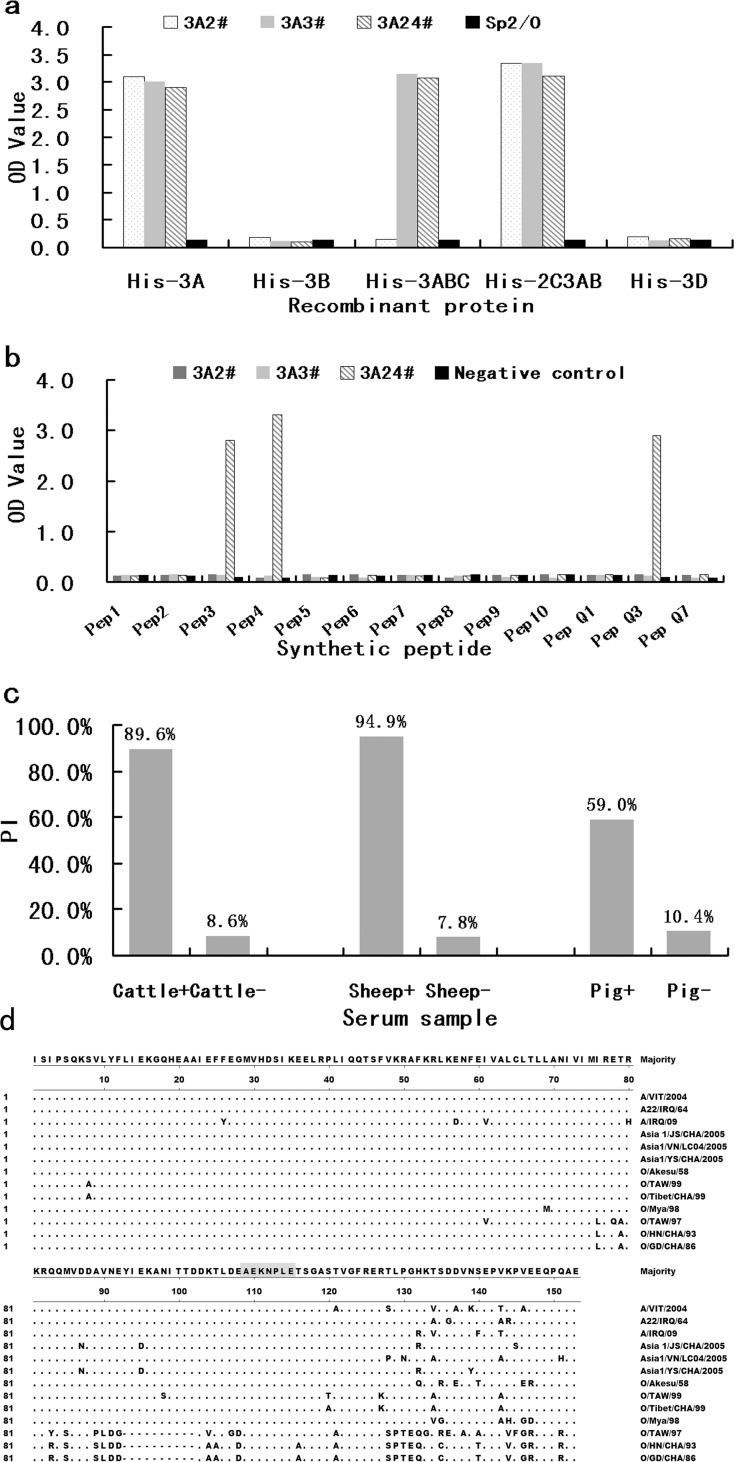
Characteristics of Mabs. (a) Reactivity of Mabs with different recombinant non-structural proteins. ‘Sp2/0’ as negative control (b) Identification of the NSP 3A Mab epitope by performing a peptide ELISA. ‘Negative control’ is the supernatant of Sp2/0 cell (c) Blocking the binding of a Mab to 2C3AB with sera from infected animals. (d) Conserved epitopes in FMDV NSP 3A.

Three Mabs, named 3A2, 3A3, and 3A24, were prepared in this study. The isotypes of the 3 Mabs were found to be IgG2a for 3A2 and 3A3, and IgG2b for 3A24 by a Mouse Monoclonal Antibody Isotyping Kit (ThermoFisher Scientific product). The results of an indirect ELISA showed that 3A3 and 3A24 specifically bound to recombinant proteins His-3A, His-3ABC, and His-2C3AB; 3A2 only bound to recombinant proteins His-3A and His-2C3AB, but not His-3ABC; none of the Mabs reacted with His-3B or His-3D; the supernatants from the Sp2/0 cell did not react with any recombinant proteins (Fig 1A). These results indicate that the 3 Mabs recognize different epitopes located in protein 3A. A peptide-screening ELISA showed that 3A2 and 3A3 did not react with any peptides located in aa 71–143 of the 3A protein (O/HN/CHA/93); thus, it is likely that they recognize epitopes either located in aa 1–70 of the 3A protein or a conformational epitope. Mab 3A24 reacted with peptides A3, A4, and Q3 (Fig 1B), which share a common “AEKNPL” sequence, indicating that the “AEKNPL” sequence was the core motif involved in 3A24 Mab recognition. As the recombinant virus (r-HN/3A93–143) deleted 93–143 aa in 3A cannot be recognized by Mab 3A24 by indirect immunofluorescent assay [9], we can conclude that 3A24 Mab is specific targeting to epitope “AEKNPLE”.

The results of the blocking ELISA showed that sera from FMDV-infected cattle, sheep, and swine could block Mab 3A24 binding to NSP 2C3AB (Fig 1C). Based on these results, we concluded that Mab 3A24 recognized a native epitope located in the 3A protein.

### Analysis of the B cell epitopes in the FMDV 3A Protein

Sequence alignment revealed a 10-aa deletion (^92^EYIEKANITT^101^; O/Tibet/CHA/99 sequence) in the NSP 3A protein in the Cathay topotype of type-O FMDV, and relatively high variations were observed between aa 76–153. However, the “AEKNPLE” epitope was well conserved among different serotypes or lineages of FMDV (Fig 1D). Thus, we can conclude that 3A24 recognized a conserved and native epitope located in the 3A protein.

### Determination of the cutoff PI value, sensitivity, and specificity of the 3A-Mab-bELISA test

**Fig 2:**
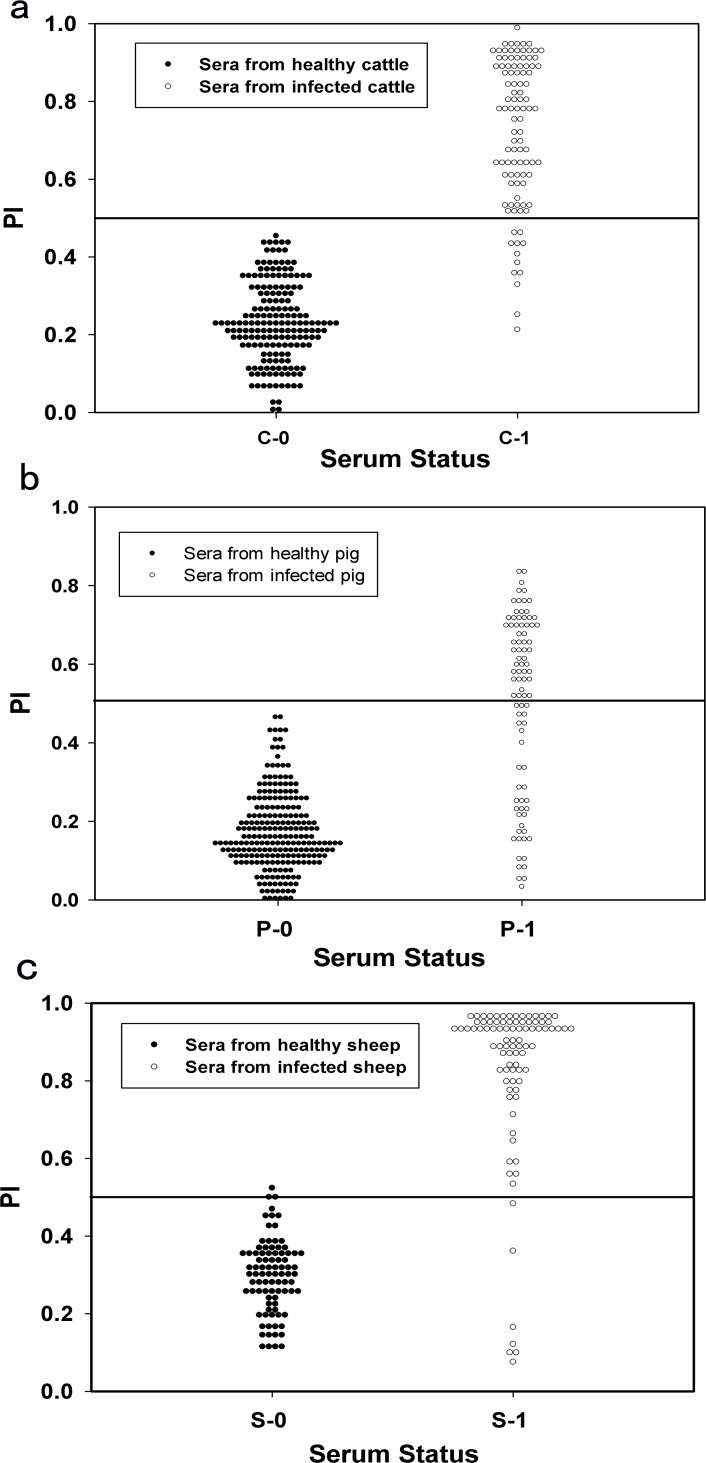
Distribution of PI values obtained by the 3A-Mab-bELISA test. (a) ‘C-0’ denotes sera from healthy cattle (84 sera from unvaccinated healthy cattle and 99 sera from healthy vaccinated cattle) and ‘C-1’ denotes infected sera (100 sera from infected cattle), (b) ‘P-0’ denotes sera from healthy pig (88 sera from unvaccinated healthy pig, 176 sera from healthy vaccinated pig) and ‘P-1’ denotes infected sera (88 sera from infected pig), and (c) ‘S-0’ denotes sera from healthy sheep (88 sera from unvaccinated healthy sheep) and ‘S-1’ denotes infected sera (88 sera from infected sheep).

Next, we aimed to determine the cutoff PI value for the 3A-Mab-bELISA test that gives high sensitivity and specificity for the method. To this end, 535 sera from uninfected animals (sera group Ⅰand Ⅱ) and 276 sera from FMDV-infected animals (sera group Ⅲ) were tested by the 3A-Mab-bELISA tests. The PI distributions are shown in Fig 2A–2C for cattle, sheep, and swine, respectively. Table 2 shows the sensitivity and specificity of the method at different cutoff values in 3 animal species. We observed high specificities of 100% (183/183), 96.6% (85/88), and 99.6% (263/264) and sensitivities of 88% (88/100), 93.2% (82/88), and 61.4% (54/88) for non-infected and infected cattle, sheep, and swine sera, using a cutoff PI value of 50%. Therefore, a PI of 50% was defined as a key criterion of the assay. To validate the assay, the mean OD450 of the negative control must be >1.0, and the mean PIs of the positive and weak-positive controls must be >70% and 50%, respectively.

**Table 2:** Specificity and sensitivity of 3A-Mab-bELISA at different cutoff values.

Species	Cutoff value	Specificity	Specificity	Sensitivity
		Naive animals	Vaccinated animals	Infected animals
Cattle	45%	97.6% (82/84)	100% (99/99)	92% (92/100)
	50%	100% (84/84)	100% (99/99)	88% (88/100)
	55%	100% (84/84)	100% (99/99)	79% (79/100)
swine	45%	98.9% (87/88)	98.9% (174/176)	68.2% (60/88)
	50%	100% (88/88)	99.4% (175/176)	61.4% (54/88)
	55%	100% (88/88)	99.4% (175/176)	54.5% (48/88)
sheep	45%	92% (81/88)	NA	93.2% (82/88)
	50%	96.6% (85/88)	NA	93.2% (82/88)
	55%	100% (88/88)	NA	90.9% (80/152)

NA: not applicable, no sera samples with clear background are available.

### Evaluating the performance of the 3A-Mab-bELISA test

**Fig 3:**
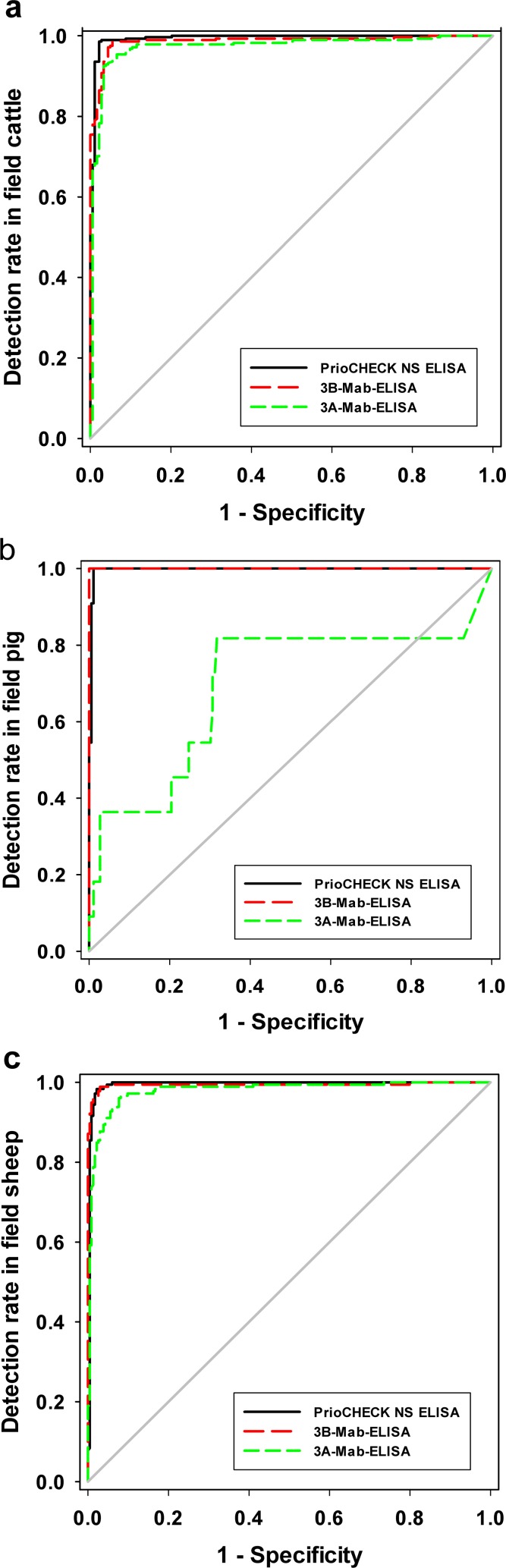
ROC curves analysis of the consistency of three blocking ELISAs using test sera from field animals. (a) Sera from 464 field cattle, (b) sera from 197 field pigs, and (c) sera from 413 field sheep.

To assess the performance of the 3A-Mab-bELISA test, panels of sera from different origins were tested by the 3B-Mab-bELISA and PrioCHECK^®^ FMDV-NS ELISA tests. The results of the 3A-Mab-bELISA test were highly consistent with 3B-Mab-bELISA and PrioCHECK^®^ NS ELISA tests for detecting sera samples from uninfected, infected, and field animals. The overall coincident rates were 94.3% (1778/1885) between the 3A-Mab-bELISA and PrioCHECK^®^ NS ELISA tests, and 93.7% (1767/1885) between the 3A-Mab-bELISA and 3B-Mab-bELISA tests, respectively. The results from the field samples showed the 3A-Mab-bELISA test was more sensitive than other 2 blocking ELISA with sheep samples, but less sensitive with pig samples (Table 3). From the ROC curves generated using results from field cattle and sheep (Fig 3A and 3C) it is clear that there are no differences between three ELISAs with respect to the detection rate of infected animals. However, the differences are significant between 3A-Mab-bELISA and other two ELISAs (*p*<0.01) for field samples from pig (Fig 3B, Table 4).

**Table 3:** Comparison of the concordance rates between the 3A-Mab-bELISA and the 3B-Mab-ELISA or the PrioCHECK NS ELISA.

Sera origin	3A-Mab-bELISA	3B-Mab-ELISA	PrioCHECK NS ELISA
	Pos./Total	Pos./Total(coincident No.)	Pos./Total(coincident No.)
Infected cattle ^a^	88/100	88/100 (92)	88/100 (92)
Infected swine ^b^	54/88	46/88 (71)	53/88 (80)
Infected sheep ^c^	82/88	82/88 (86)	84/88 (86)
Vaccinated cattle	0/99	0/99 (99)	0/99 (99)
Vaccinated swine	0/176	0/176 (176)	0/176 (176)
Uninfected cattle	0/84	0/84 (84)	0/84 (84)
Uninfected sheep	0/88	1/88 (87)	1/88 (87)
Uninfected swine	0/88	0/88 (88)	0/88 (88)
Field cattle	281/464	284/464 (427)	281/464(429)
Field sheep	200/413	176/413 (373)	177/413 (374)
Field swine	9/197	11/197 (184)	13/197 (183)
Coincident rate	/	93.7% (1767/1885)	94.3% (1778/1885)

^a^ sera from cattle infected with Asia 1/JS/CHA/05 or O/Tibet/CHA/99 FMDV collected between 10–28 DPI

^b^ sera from swine infected with O/ Tibet/CHA/99 collected between 3–11 DPI.

^c^ sera from sheep infected with Asia 1/JS/CHA/05 collected between 4–365 DPI.

**Table 4:** Pairwise comparison of the three ELISAs based on areas under the ROC curves.

Animal	Tests	PrioCHECK ELISA	3B-Mab-bELISA
		(*P* value)	(*P* value)
Field cattle	3A-Mab-bELISA	* (0.017)	- (0.128)
	3B-Mab-bELISA	- (0.223)	
Field pig	3A-Mab-bELISA	**(0.004)	** (0.004)
	3B-Mab-bELISA	- (0.282)	
Field sheep	3A-Mab-bELISA	- (0.055)	- (0.073)
	3B-Mab-bELISA	- (0.958)	

95% confidence interval; (-) Not significant

* significant *P*<0.05

** significant *P*<0.01

**Fig 4:**
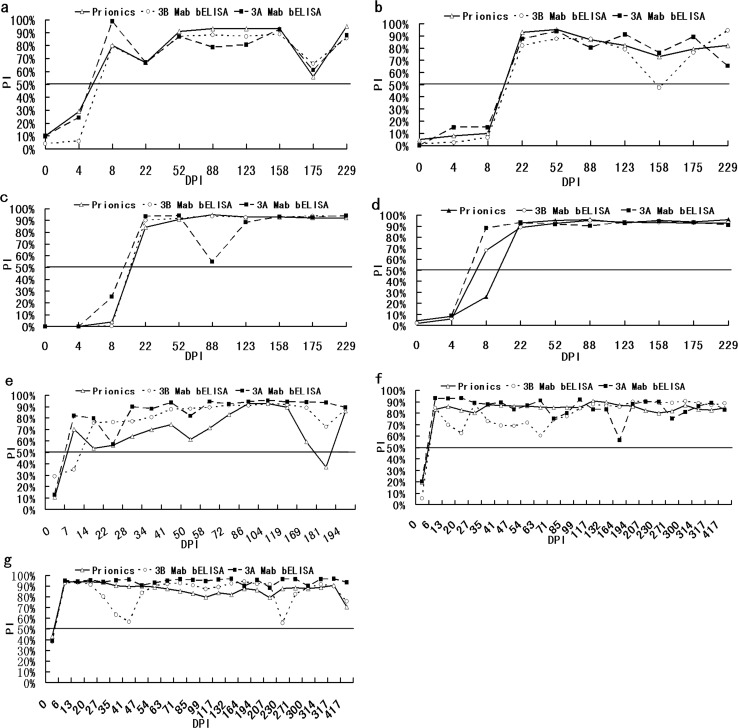
Detection with serum antibodies collected sequentially from animals infected with FMDV by the 3A-Mab-bELISA, 3B-Mab-bELISA and PrioCHECK^®^ NS ELISA tests. (a)–(d) Results from cattle No. 4009, 0738, 4017, and 4004, respectively. (e) Results from 1 pig infected with FMDV; and (f)–(g) show the results from two sheep No. 40 and 54 infected with FMDV.

The results of 104 sequential serum samples from 4 cattle (n = 40), 2 sheep (n = 48), and 1 swine (n = 16) infected with FMDV in the three test are shown in Fig 4A–4G. Of the 40 serum samples from 4 infected cattle, 39 samples yielded identical results in the three tests (Fig 4A–4D). Only 1 sample, taken from animal no. 4004 at 8 DPI, showed a positive result with the 3B-Mab-bELISA and 3A-Mab-bELISA tests, but not the PrioCHECK^®^ NS ELISA test (Fig 4D). Of the 16 sera from 1 infected swine, there was agreement between all assays for 14/16 samples (Fig 4E). With all 48 serum samples from 2 infected sheep, identical results were observed with each of the 3 methods (Fig 4F and 4G).

### Detecting serum samples from animals inoculated with the NSP 3A epitope-deleted marker virus by the 3A-Mab-bELISA test

**Fig 5:**
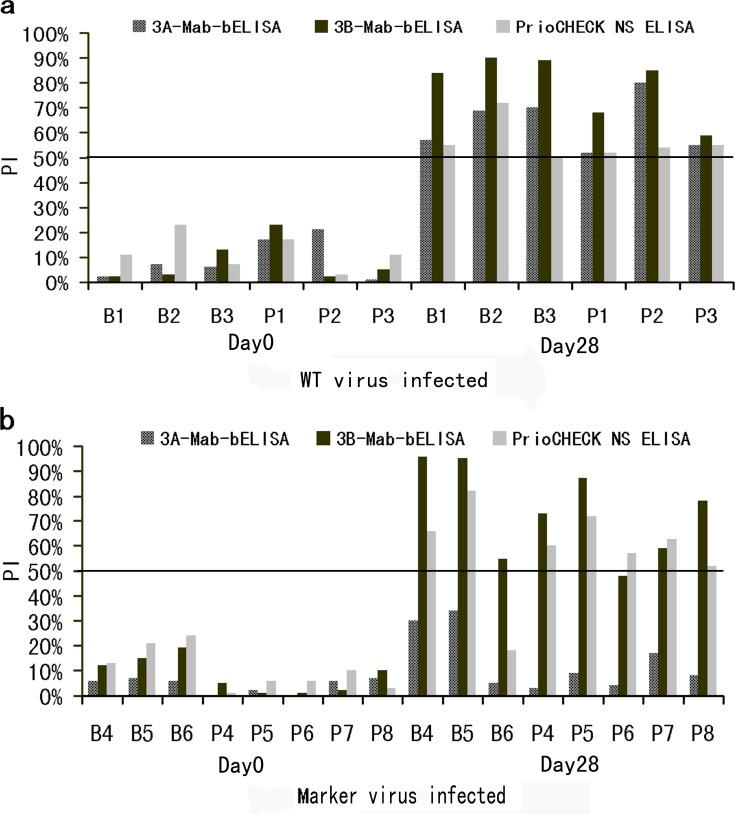
Detection of serum antibodies collected before and after infection with wild type virus and marker virus at 28 DPI, by the 3A-Mab-bELISA, 3B-Mab-bELISA and PrioCHECK^®^ NS ELISA tests. (a) Results showing differential antibody responses in animals before and after infection with wild type FMDV. (b) Results of differential antibody responses in animals before and after infection with the marker virus.

As shown in Fig 5A and 5B, all sera (n = 14) from animals before inoculation with the wild type or marker virus showed negative results by 3 blocking-ELISA tests, and all six animals infected with wild type FMDV shown positive results at 28 DPI in the 3 ELISAs. The sera from the eight animals inoculated with the marker virus given positive results at 28 DPI by the 3B-Mab-bELISA and PrioCHECK^®^ NS ELISA tests, except that 1 sample from cattle B6 at 28 DPI showed a negative result by the PrioCHECK^®^ NS ELISA. In contrast, all sera from marker virus-inoculated animals at 28 DPI were negative by the 3A-Mab-bELISA test. These results demonstrated that 3A-Mab-bELISA can serve as a companion test for the 3A epitope “AEKNPLE” deleted marker virus vaccine.

## Discussion

In developing countries, the control and eradication of foot-and-mouth disease mainly relies upon vaccination. However, how to effectively distinguish infected animals from vaccinated animals is an important challenge because this is the basis for detecting and culling potentially virus carrier animals. A major problem stems from the unpurified vaccine antigen with residual NSPs, which will interfere with DIVA test based on detecting NSP antibodies. Although the sensitivity of the NSP-based DIVA test is decreased under vaccinated condition, it remains the most useful method for evaluating the infection status on a herd level [20–22]. In China, it is not officially required that residual NSPs be removed from the FMD vaccine antigen, although virus suspension culture and antigen production techniques have been improved greatly to reduce the level of NSP. Using an unpurified vaccine causes further problems for disease serological surveillance and evaluation of the infection status of animals. Thus, research on negative-marker FMD virus vaccines and its companion DIVA test are important for reducing vaccine costs and resolving the above difficulties for DIVA purposes.

Identification and screening of a conserved and dominant epitope is important for the development of negative marker vaccine and the corresponding DIVA test. Many linear B cell epitopes in FMDV NSPs have been identified previously [4]; however, conserved and immunodominant epitopes that can be deleted or modified to generate a negative-marker virus are rare. In this study, one Mab 3A24 was found to recognize the conserved epitope ^109^AEKNPLE^115^ of NSP 3A. This epitope can be deleted by a reverse-genetic operation to generate a negative-marker virus, which can be used to develop FMD marker vaccine [9]. Binding of Mab 3A24 to NSP 2C3AB could be blocked by serum samples derived from the animals infected with different serotypes of FMDVs, which demonstrated that Mab 3A24 recognized a native epitope in 3A. Based on these results, we conclude that the residues of ^109^AEKNPLE^115^ is a conserved and dominant epitope in 3A and is a potential candidate site for developing negative-marker viruses and vaccines against different FMDV serotypes.

To develop the blocking ELISA, the 2C3AB protein was captured by a polyclonal antibody to 2C coated on an ELISA plate, which facilitated renaturation of the prokaryotically expressed 2C3AB protein and improved the accuracy of DIVA testing. Using this ELISA format may contribute to improve test performance. The cutoff PI value of the 3A-Mab-bELISA test was determined to be 50% by testing panels of sera from different hosts, which resulted in a high sensitivity and specificity. Based on the cutoff value of 50%, specificities of 100% (183/183), 100% (88/88), and 100% (264/264) and sensitivities of 88% (88/100), 93.2% (82/88), and 61.4% (54/88) were observed with non-infected and infected cattle, sheep, and swine sera, respectively. The 3A-Mab-bELISA test also showed good performance when compared with the other 2 blocking ELISAs. The coincident rates with the commercial PrioCHECK^®^ NS ELISA and 3B-Mab-bELISA were 94.3% and 93.7%, respectively, these results indicating that the 3A-Mab-bELISA test is a competent method for DIVA.

Results from the field samples showed that the 3A-Mab-bELISA test was more sensitive than other 2 bELISA with sheep samples, but less sensitive with pig samples. Using pair-wise comparison (ROC analysis) of data from field cattle and sheep, no significant difference was evident between three ELISAs. However, 3A-Mab-bELISA has significantly lower detection rate in field pig than the other 2 bELISA. This difference may imply that the dominant epitopes in NSP 3A and 3B induced varied antibody responses in different animal species. These results may further indicate that a second ELISA test is necessary to improve the accuracy of the DIVA test [21, 22].

The serum-detection results showed that the animals infected with 3A epitope-deleted marker virus did not develop a measurable antibody against the deleted epitope, while animals inoculated with the wild type virus induced high-level antibody against the corresponding epitope at 28 DPI. This result showed a new strategy to create a negative-marker virus vaccine by deletion of the ^109^AEKNPLE^115^ epitope in NSP 3A. The 3A-Mab-bELISA test described here can serve as a matching test for the 3A epitope-deleted marker virus vaccine. However, some virus strains may be attenuated after removal of the ‘AEKNPLE’ epitope in 3A, which will influence the virus antigen yield in cell culture. So, further works are necessary to identify the critical aa sites that can completely abolish Mab 3A24 binding to this epitope.

In conclusion, a Mab targeting the NSP 3A “AEKNPLE” epitope was prepared in order to develop a DIVA test for FMDV surveillance. This epitope can be modified or deleted by a reverse-genetic operation to create a modified virus, which can be used to produce a negative marker vaccine. The ELISA test described here is a potential companion DIVA method for an NSP 3A epitope-deleted FMDV marker vaccine, and this approach may greatly improve the efficacy of FMD control and eradication efforts in the future.
